# Recurrent obstructive sleep apnea precipitated by vagus nerve stimulator despite weight loss and uvulopalatopharyngoplasty

**DOI:** 10.1002/epd2.20334

**Published:** 2025-01-28

**Authors:** Derek C. P. Fisk, Marcus C. Ng

**Affiliations:** ^1^ Department of Psychiatry, Max Rady College of Medicine University of Manitoba Winnipeg Manitoba Canada; ^2^ Section of Neurology, Department of Internal Medicine, Rady Faculty of Health Sciences University of Manitoba Winnipeg Manitoba Canada

**Keywords:** epilepsy, obstructive sleep apnea, seizure, sleep case report, vagus nerve stimulator

**Key points**

- Obstructive sleep apnea (OSA) is highly prevalent in patients with epilepsy.
- In this clinical vignette, a patient's previously cured OSA re‐emerges following vagus nerve stimulator (VNS) implantation.
- VNS may precipitate OSA even in those who have been cured by lifestyle and surgical interventions.
- Patients with VNS should be carefully monitored for OSA and treated when it occurs to ensure optimal device tolerability and seizure control.

Obstructive sleep apnea (OSA) is highly prevalent in epilepsy and significantly impacts severity and treatment effectiveness.^1^, ^2^ Vagal nerve stimulators (VNS) are commonly used to treat drug‐resistant epilepsy (DRE) and are associated with an increased risk of OSA.^3^, ^4^, ^5^, ^6^, ^7^, ^8^, ^9^, ^10^ Herein, we report a patient with DRE whose OSA recurred after VNS implantation despite a previous uncomplicated uvulopalatopharyngoplasty (UPPP) and significant weight loss.

In 1995, a 29‐year‐old right‐handed male developed temporal lobe epilepsy (TLE) 2 years after a bout of viral meningitis. His seizures were refractory to multiple anti‐seizure medications (ASM), cannabidiol, and a ketogenic diet. In 2003, he was diagnosed with OSA on a home sleep study (records unavailable) despite prior tonsillectomy. In 2005, he underwent UPPP and went on to lose 64 pounds, lowering his BMI from 34.7 to 25.8 kg/m^2^. All OSA symptoms resolved without the need for continuous positive airway pressure (CPAP). In 2013, he was referred to an epilepsy surgical center where he first received left lesional corticoamygdalectomy as part of a two‐stage approach to minimize risks of memory impairment from a resection in the dominant hemisphere. Nevertheless, a larger resection was later planned if seizures persisted.

He continued experiencing focal unaware seizures with gustatory aura, most commonly upon awakening and triggered by sleep deprivation. These seizures were confirmed by video‐EEG telemetry in 2016 but he initially declined pursuing the second stage of the two‐staged approach recommended by the epilepsy surgical center due to persistent fears of memory impairment. Instead, he received a VNS (SenTiva™ M1000) in 2019. After the procedure, the dosing of his Lamotrigine, Topiramate, and Clobazam (started in 2010) remained unchanged. The device's autotitration schedule was followed to an output current (OC) of 1 mA. Settings were then increased every 2 months to optimize seizure control. During autotitration, he reported recurrent hoarseness and shortness of breath (OC: 0.25 mA) and throat tightness (OC: 1.0 mA). These symptoms occurred for less than a minute every 5 min, consistent with respective VNS signals on and off‐times. At the settings displayed in Table S1 (Supporting information), he started experiencing unrefreshing sleep, increased daytime somnolence, and worsened snoring according to his partner.

In 2021, a home sleep study was performed at a time when OSA symptoms remained unchanged despite OC reduction from 1.75 to 1.625 mA. The study (Figure 1) confirmed moderate OSA. Sleep‐disordered breathing events occurred at an oddly consistent frequency of three to four times every 10 min, closely corresponding to the 3‐min VNS cycle. Trials of dental appliances and CPAP failed. In 2022, due to seizure persistence and the side effects described above, he requested VNS removal. Further trials of reduced settings were declined. VNS explantation resulted in improved sleep, reduced daytime somnolence, and a cessation of snoring according to his partner. In 2023, he underwent stereo‐EEG followed by left anterior temporal lobectomy, which has rendered him seizure‐free for 2 years.

**FIGURE 1:**
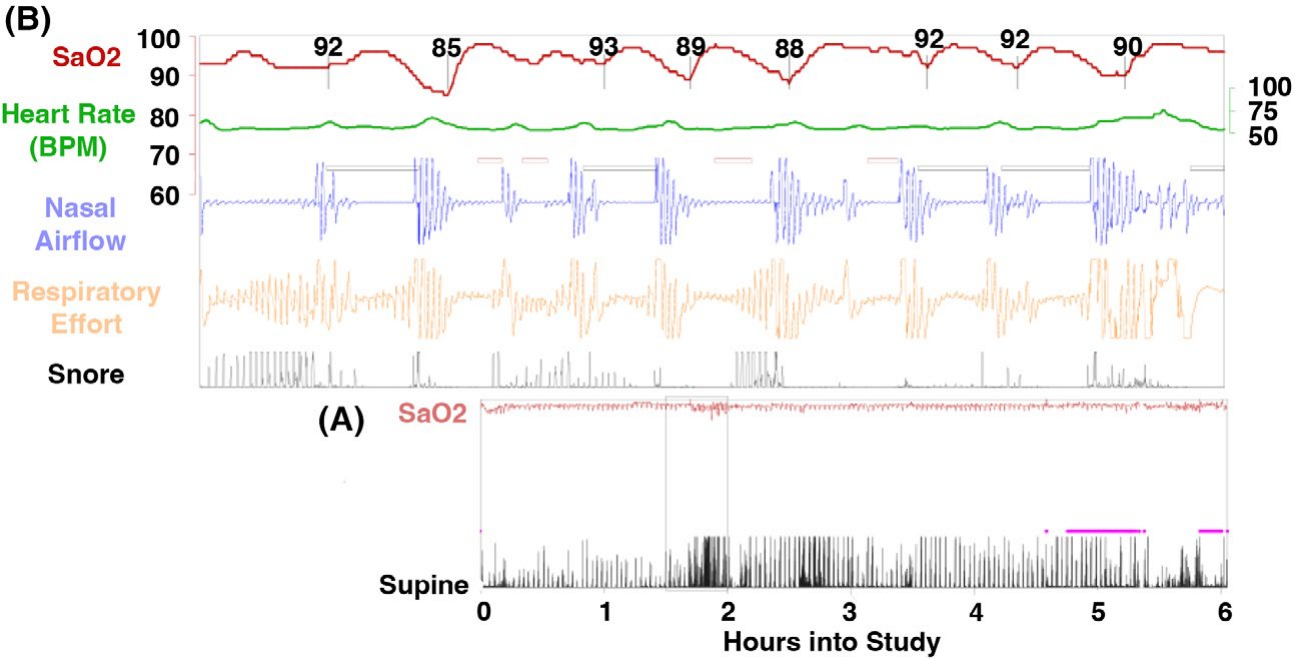
Level 3 remmers sleep recorder home sleep study to investigate sleep disturbance post‐vagus nerve stimulator implantation. Performed while the patient was on all home medications. The duration of the study was 6.1 h. The mean oxygen saturation was 95.4%. The lowest oxygen saturation was 85%. The total Apnea‐Hypopnea Index was 18.5 events per hour. The study was interpreted by a board‐certified sleep specialist physician. (A) Total study. The first horizontal line represents oxygen saturation (SaO2). The second horizontal line represents the supine sleeping position. Vertical bars at the bottom represent snoring. (B) Inset of vertical rectangles from total study. The first horizontal line represents oxygen saturation (SaO2). The second horizontal line is heart rate (BPM: beats per minute). The third horizontal line is nasal airflow. Horizontal bars above the third horizontal line represent apneas or hypopneas. The fourth horizontal line is respiratory effort. Vertical bars at the bottom represent snoring.

Limitations include the sleep study before VNS occurring over a decade prior to implantation. Furthermore, no sleep study was performed after VNS explantation such that OSA resolution was based clinically. Similarly, timing of VNS activation relative to apneic events was not recorded during the sleep study. However, the strongly coincidental frequency of VNS activation and apnea raised concern that his VNS was directly responsible for OSA relapse. Moreover, OSA recurred despite significant weight loss. As obesity is a significant OSA risk factor,^11^, ^12^ the significant BMI reduction at the time of OSA recurrence points toward an alternate explanation for relapse such as his VNS.

Our case agrees with a growing body of epilepsy literature that indicates a likely role of VNS in precipitating OSA.^13^ Indeed, OSA prevalence in adult patients with DRE increases from 16.7% to 37.5% after VNS implantation.^10^ Many hypothesized mechanisms exist, including vocal cord paresis, supraglottic muscle collapse,^14^ or brainstem‐mediated changes in respiration.^7^ The OC at which OSA recurred (1.75 mA) is congruent with observations that OSA most commonly occurs at and above 2 mA,^10^ but can occur as low as 1.25 mA.^15^, ^16^ Similarly, the OC and duty cycle (25%) at OSA recurrence is corroborated by Fahoum et al.'s^17^ observation that tolerability and probability of response decreases above an OC of 1.61 mA or duty cycle over 17.1%.

Our case highlights the potential ability of VNS to recapitulate previously resolved OSA despite significant lifestyle and surgical intervention such as UPPP. Consequently, clinicians should hold a high index of suspicion and low threshold to screen for OSA in such patients, even those who have undergone significant weight loss or OSA surgery. Given that OSA adversely impacts seizure control,^1^ OSA occurrence after VNS implantation could interfere with a patient's ability to tolerate VNS titration to optimal anti‐seizure settings. In such cases, significant efforts to treat OSA may improve seizure control directly by removing OSA as a seizure‐provoking factor, and indirectly by helping patients reach maximally effective VNS settings.

## CONFLICT OF INTEREST STATEMENT

MCN receives publishing royalties from Demos Medical Publishing. He also receives speaking honoraria from and is on the advisory boards for Eisai Canada and UCB Canada. He is on the advisory board for Paladin Canada. All honoraria were donated to the local hospital charity foundation.

**Test yourself**

1. Which of the below options represents a mechanism by which VNS are believed to cause OSA in some patients?
  - Stimulation of cholinergic nerves leading to bronchoconstriction.
  - Narrowing of the nasal airways leading to obstruction.
  - Relaxation of the epiglottis resulting in tracheal obstruction.
  - Vocal cord paresis leading to airway obstruction.
2. In patients who receive VNS for DRE, OSA has been most frequently observed in patients with output currents that are:
  - ≤1.00 mA.
  - 1.50 –2.00 mA.
  - ≥2.00 mA.
  - ≥2.50 mA.
3. Which of the following was discussed as a reason to treat OSA when it occurs in patients being treated with a VNS?
  - VNS have been clearly shown to improve sleep quality in patients with epilepsy.
  - Treating OSA can facilitate further titration to optimal VNS settings.
  - Improvements in mood symptoms following OSA treatment result in decreased seizure frequency.
  - Snoring and intermittent airway obstruction interferes with VNS functioning.

*Answers may be found in the* *Supporting information*.

## Supporting information


Data S1.

Data S2.

Table S1.
