# Supplementary material for: Recurrent obstructive sleep apnea precipitated by vagus nerve stimulator despite weight loss and uvulopalatopharyngoplasty
Source: Epileptic Disord. 2025 Jan 28;27(2):295–8. doi: 10.1002/epd2.20334 (PMC12065122; doi:10.1002/epd2.20334)
Supplement: Supplementary file 1 — Data S1. [file EPD2-27-295-s002.docx]

**Test Yourself**

**Answers:**

1. D

2. C

3. B
