# Supplementary material for: Recurrent obstructive sleep apnea precipitated by vagus nerve stimulator despite weight loss and uvulopalatopharyngoplasty
Source: Epileptic Disord. 2025 Jan 28;27(2):295–8. doi: 10.1002/epd2.20334 (PMC12065122; doi:10.1002/epd2.20334)
Supplement: Supplementary file 2 — Data S2. [file EPD2-27-295-s003.pptx]

## Slide 1
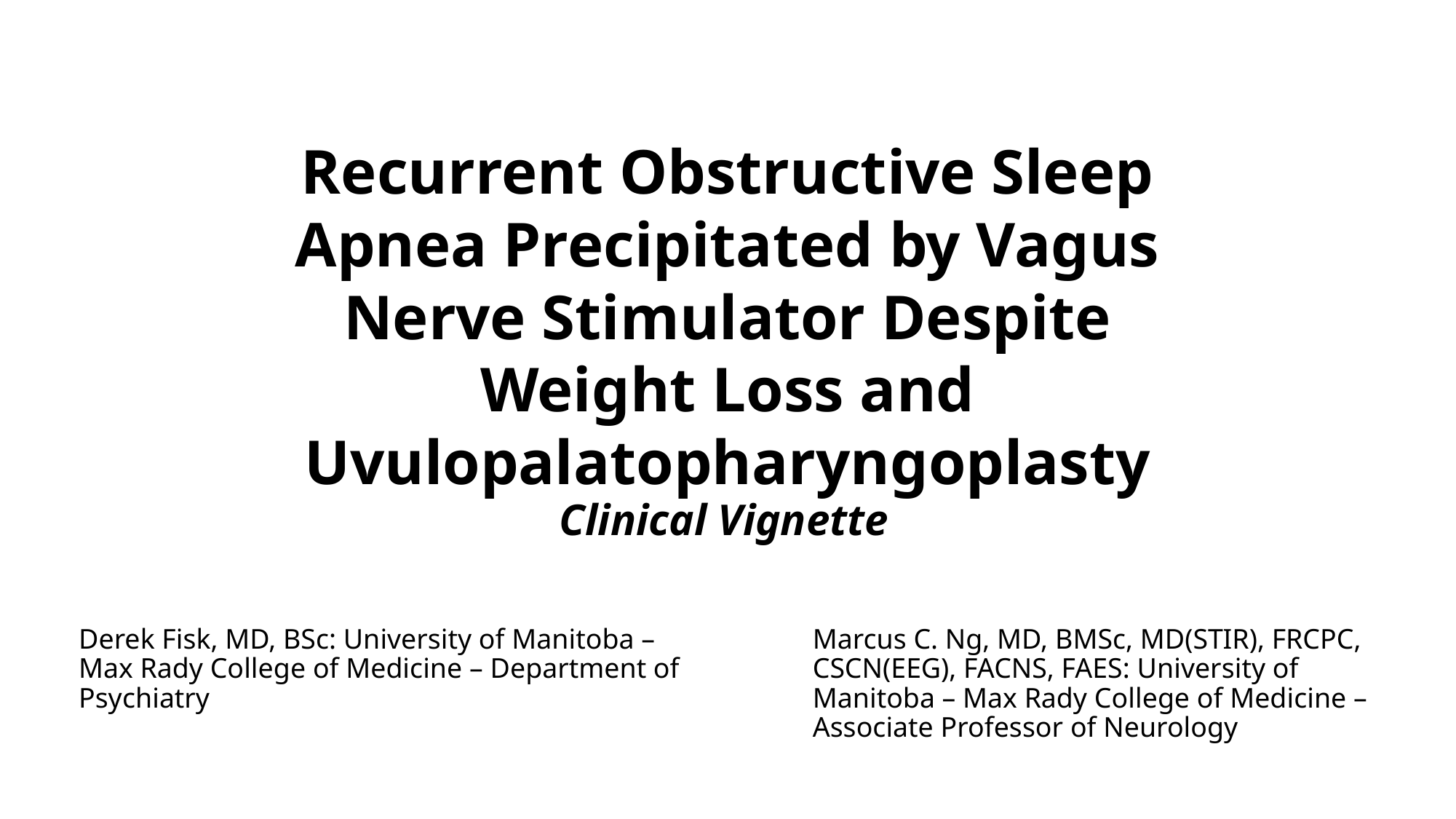

Recurrent Obstructive Sleep Apnea Precipitated by Vagus Nerve Stimulator Despite Weight Loss and Uvulopalatopharyngoplasty
Clinical Vignette
Marcus C. Ng, MD, BMSc, MD(STIR), FRCPC, CSCN(EEG), FACNS, FAES: University of Manitoba – Max Rady College of Medicine – Associate Professor of Neurology
Derek Fisk, MD, BSc: University of Manitoba – Max Rady College of Medicine – Department of Psychiatry

## Slide 2
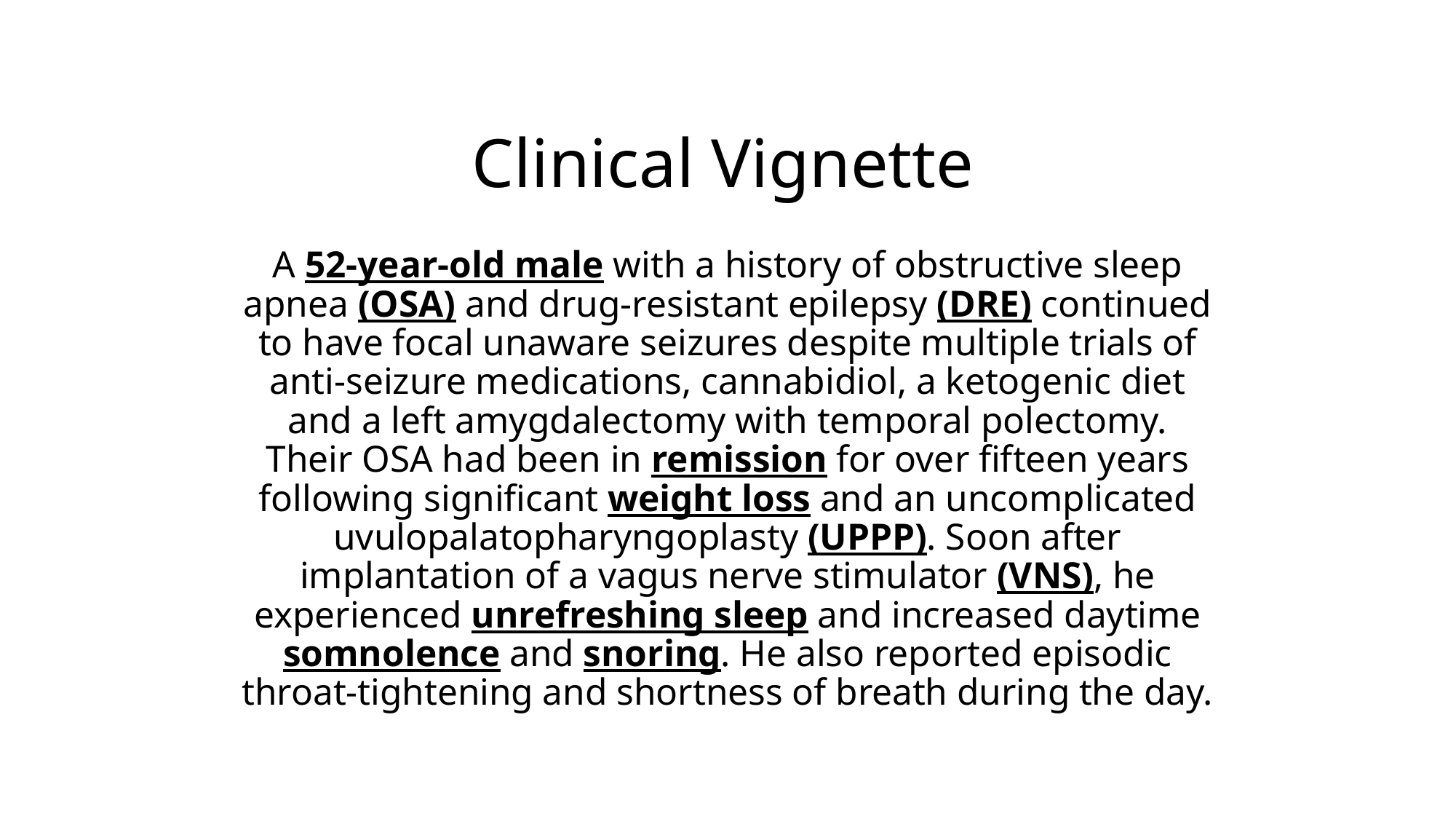

# Clinical Vignette
A 52-year-old male with a history of obstructive sleep apnea (OSA) and drug-resistant epilepsy (DRE) continued to have focal unaware seizures despite multiple trials of anti-seizure medications, cannabidiol, a ketogenic diet and a left amygdalectomy with temporal polectomy. Their OSA had been in remission for over fifteen years following significant weight loss and an uncomplicated uvulopalatopharyngoplasty (UPPP). Soon after implantation of a vagus nerve stimulator (VNS), he experienced unrefreshing sleep and increased daytime somnolence and snoring. He also reported episodic throat-tightening and shortness of breath during the day.

## Slide 3
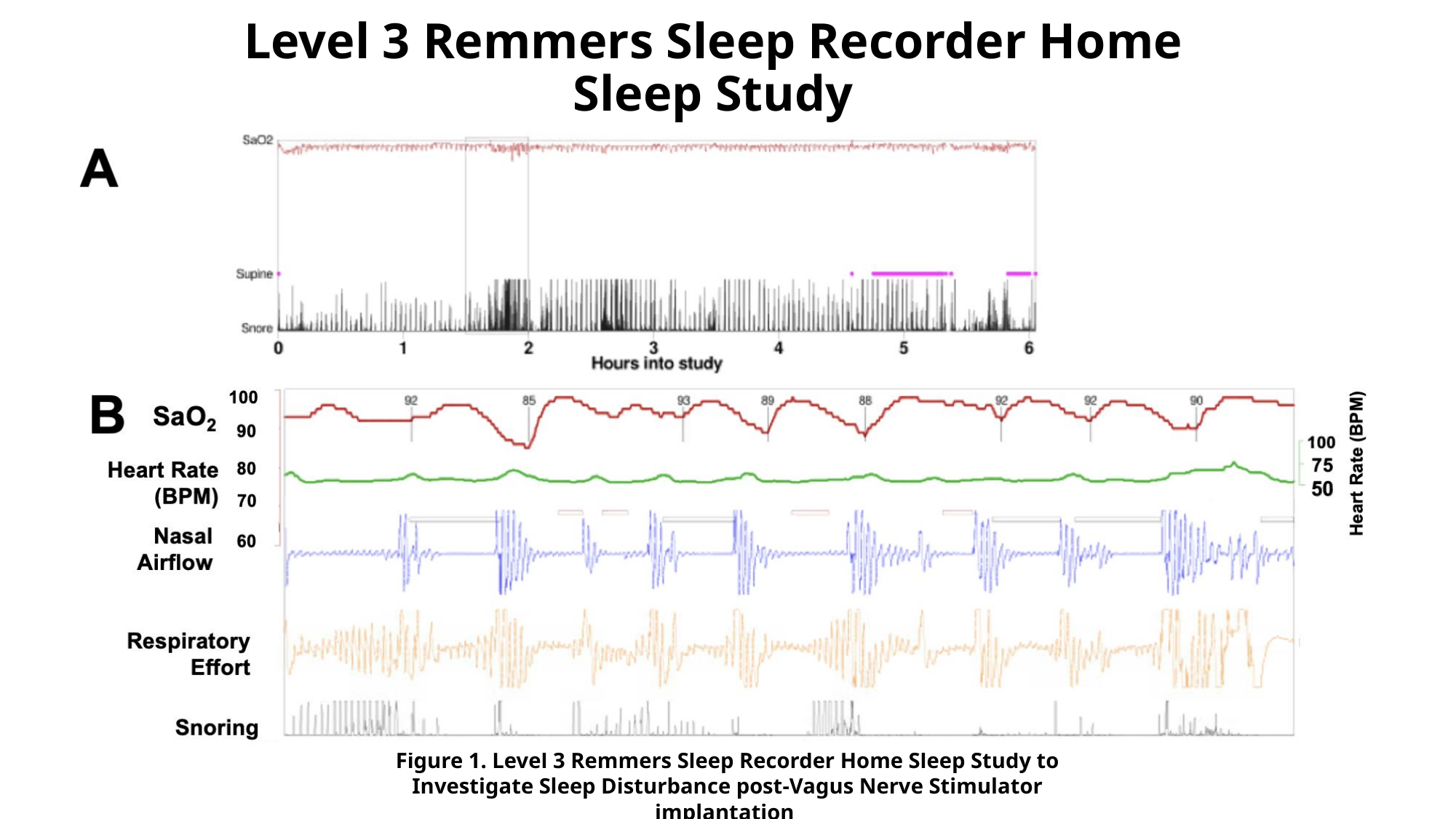

Level 3 Remmers Sleep Recorder Home Sleep Study
Figure 1. Level 3 Remmers Sleep Recorder Home Sleep Study to Investigate Sleep Disturbance post-Vagus Nerve Stimulator implantation

## Slide 4
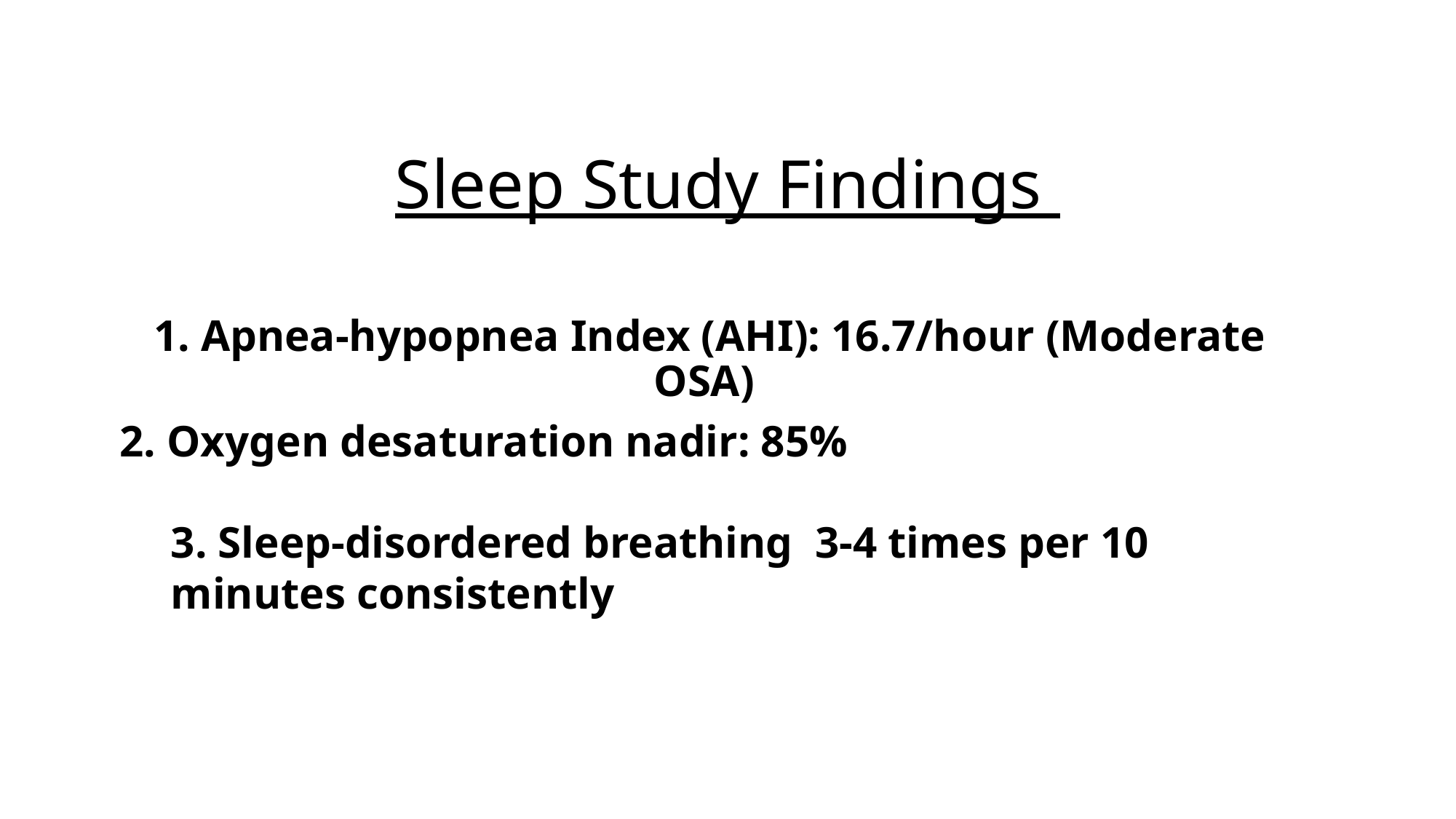

# Sleep Study Findings
1. Apnea-hypopnea Index (AHI): 16.7/hour (Moderate OSA)
2. Oxygen desaturation nadir: 85%
3. Sleep-disordered breathing 3-4 times per 10
minutes consistently

## Slide 5
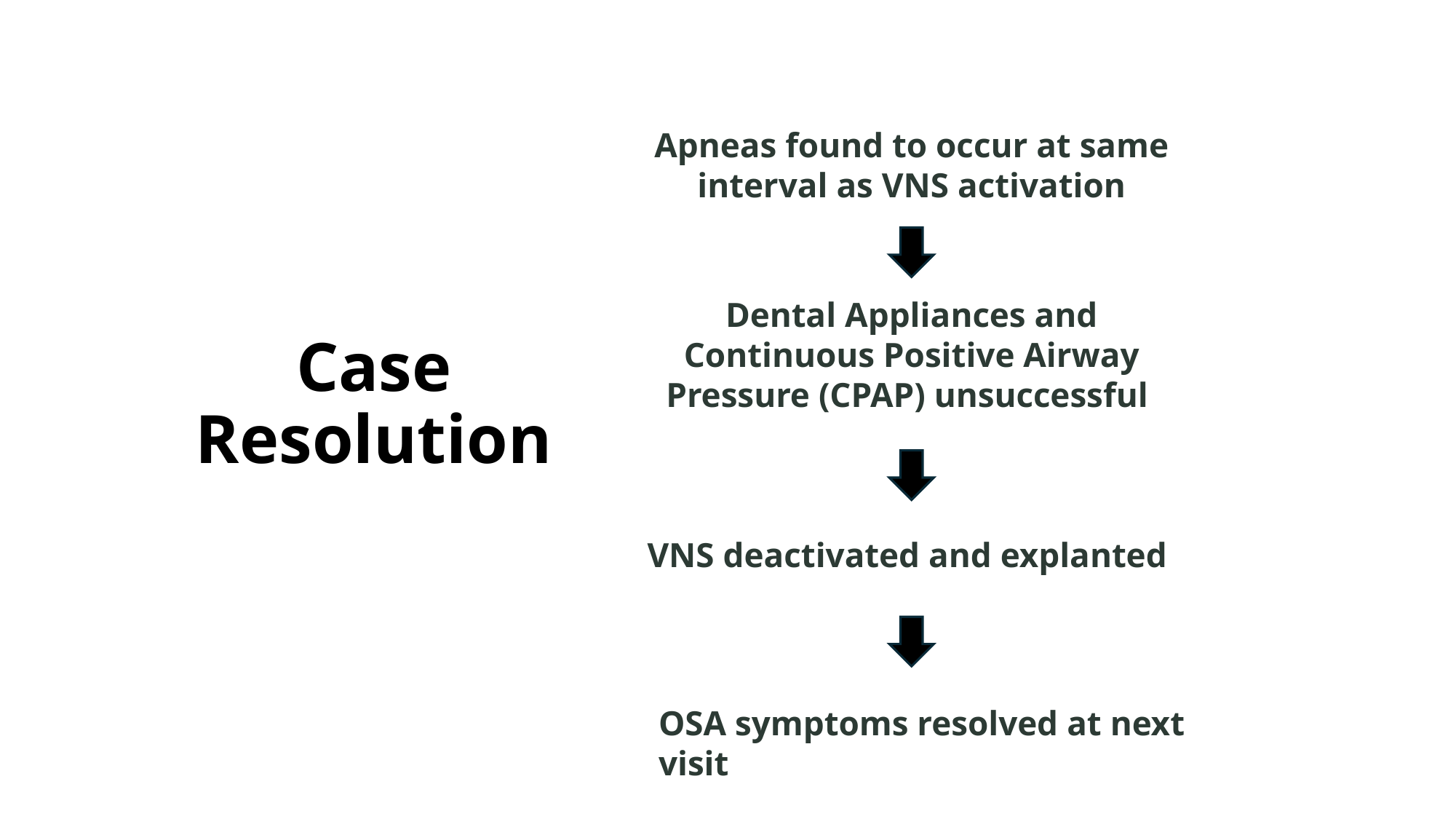

Apneas found to occur at same interval as VNS activation
# Case Resolution
Dental Appliances and Continuous Positive Airway Pressure (CPAP) unsuccessful
VNS deactivated and explanted
OSA symptoms resolved at next visit

## Slide 6
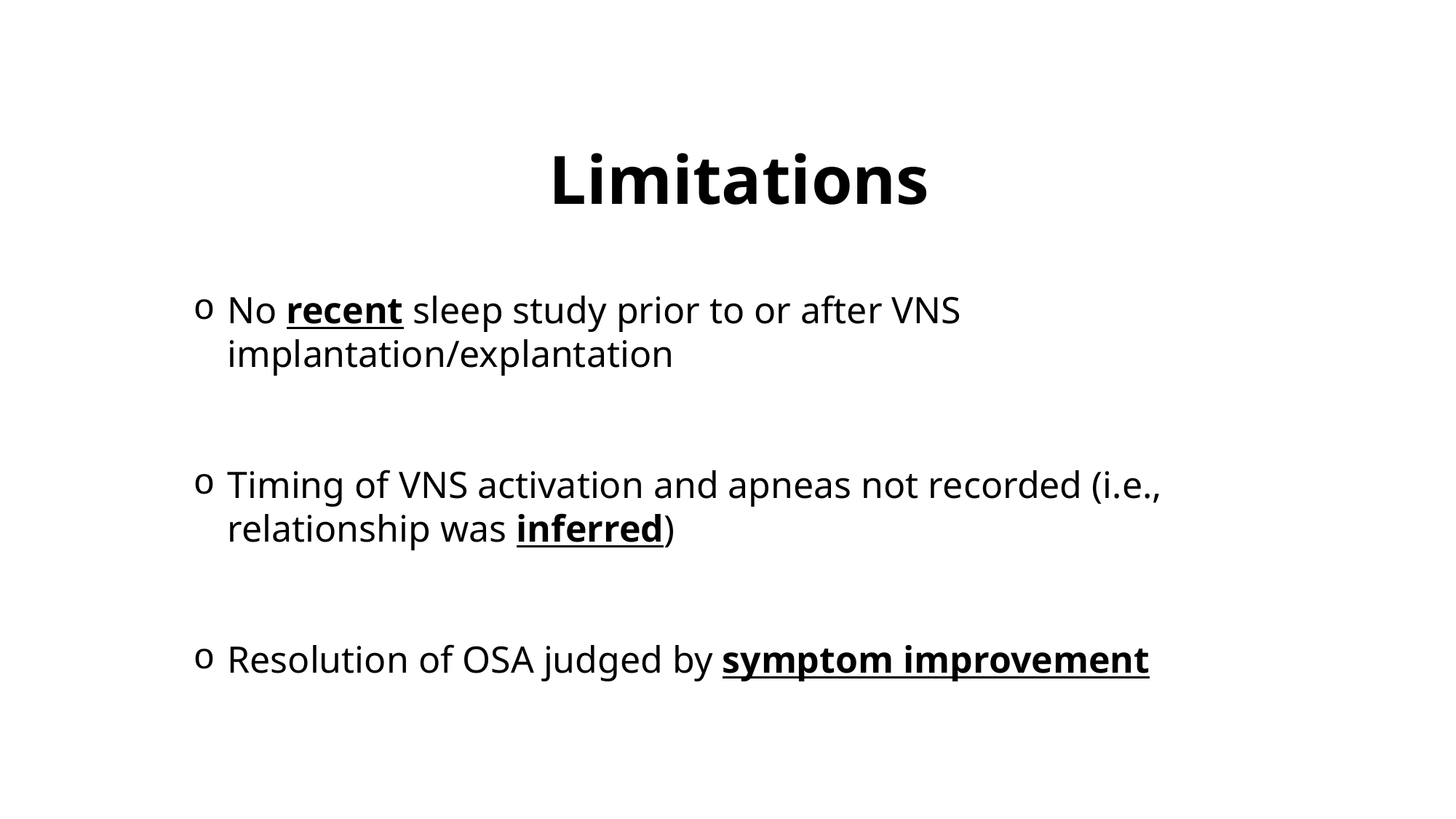

# Limitations
No recent sleep study prior to or after VNS implantation/explantation
Timing of VNS activation and apneas not recorded (i.e., relationship was inferred)
Resolution of OSA judged by symptom improvement
Limitations:
No sleep study immediately prior to or after VNS implantation/explantation
Timing of VNS activation and apneas not recorded (relationship inferred)
Resolution of OSA judged by symptom improvement.
Confounders:
OSA incidence increases linearly with age [1]
Roughly 1/3 of UPPP responders experience recurrence after 3 years [2]

## Slide 7
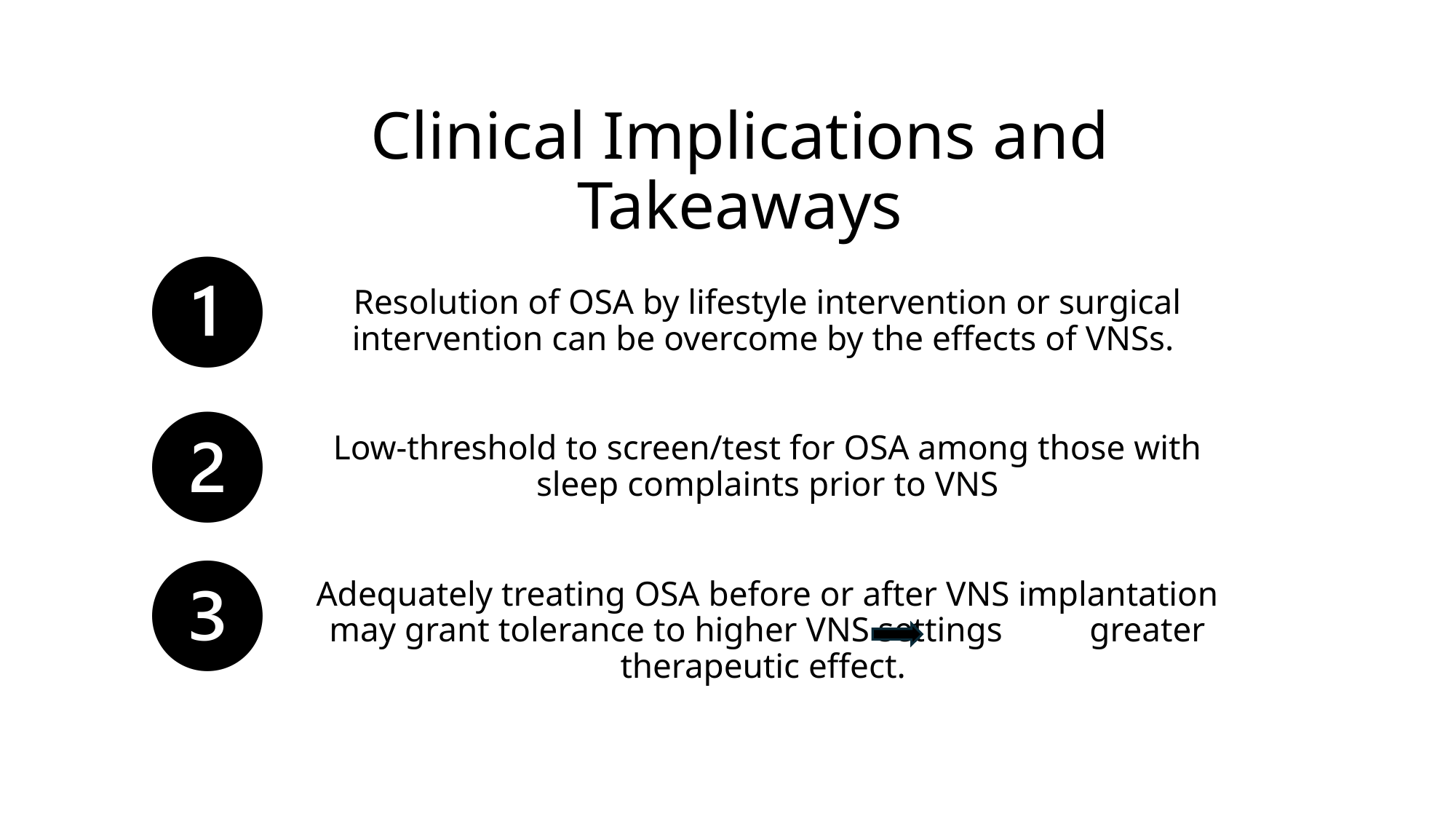

# Clinical Implications and Takeaways
Resolution of OSA by lifestyle intervention or surgical intervention can be overcome by the effects of VNSs.
Low-threshold to screen/test for OSA among those with sleep complaints prior to VNS
Adequately treating OSA before or after VNS implantation may grant tolerance to higher VNS settings greater therapeutic effect.
