# Supplementary material for: Recurrent obstructive sleep apnea precipitated by vagus nerve stimulator despite weight loss and uvulopalatopharyngoplasty
Source: Epileptic Disord. 2025 Jan 28;27(2):295–8. doi: 10.1002/epd2.20334 (PMC12065122; doi:10.1002/epd2.20334)
Supplement: Supplementary file 3 — Table S1. [file EPD2-27-295-s001.docx]

| **Table 1.** Vagus Nerve Stimulator Settings at Time of Obstructive Sleep Apnea Symptom Recurrence. | |
| --- | --- |
| **Output current** | 1.75 mA |
| **Signal frequency** | 20 Hz |
| **Pulse width** | 250 μs |
| **Signal on-time** | 30 seconds |
| **Signal off-time** | 3 minutes |
| **Magnet current** | 2 mA |
| **Magnet on-time** | 60 seconds |
| **Magnet pulse width** | 500 μs |
| **Heart rate detection setting** | 3 |
| **Auto-stimulation pulse current** | 1.875 mA |
| **Auto-stimulation pulse width** | 250 μs |
| **Auto-stimulation signal on-time**  **Duty cycle** | 30 seconds  16% |
| **Intensified follow-up indicator** | No |
